# Determinants of Urban Expansion and Spatial Heterogeneity in China

**DOI:** 10.3390/ijerph16193706

**Published:** 2019-10-01

**Authors:** Ming Li, Guojun Zhang, Ying Liu, Yongwang Cao, Chunshan Zhou

**Affiliations:** 1 Guangdong Provincial Key Laboratory of Urbanization and Geo-simulation, School of Geography and Planning, Sun Yat-sen University, No. 135, Xingang Xi Road, Guangzhou 510275, China; 2 School of Public Administration, Guangdong University of Finance and Economics, No.21 Luntou Road, Guangzhou 510320, China; 3 The Tourism College, Xinjiang University, No.1230 Yan’an Road, Urumchi 830001, China

**Keywords:** urban expansion, determinants, spatial heterogeneity, hot spot analysis, GWR, China

## Abstract

China is the world’s largest developing country and its regions vary considerably. However, spatial heterogeneity in determinants of urban expansion in prefecture-level cities have not been identified. The present study explored the spatiotemporal characteristics of Chinese urban expansion and adopted a geographically weighted regression (GWR) method to determine this spatial heterogeneity. The results indicated that China experienced massive urban expansion during 1990–2015, with urban areas growing from 4.88 × 10^4^ km^2^ to 1.06 × 10^5^ km^2^, 46.42% of which was distributed in the eastern region. The results of the GWR model revealed the spatial heterogeneity in the determinants of urban expansion. Marketization was vital for urban expansion and had a stronger impact in the developed eastern and southern regions than in the less-developed northern and western regions. Globalization and decentralization bi-directionally affected urban expansion. The constraining effects of physical factors were limited and stronger in the developing northern region than in the developed southern region. Identifying the varying determinants of urban expansion is essential for policy-making in various regions.

## 1. Introduction

China has undergone considerable urbanization since the reform in 1978. The rapid pace of urbanization has caused substantial urban expansion that has profoundly changed the global ecosystem and resulted in a multitude of environmental problems [1,2]. Besides, massive urban expansion has encroached on lots of farmland and threatened food security [3]. Restricting urban sprawl and improving land use efficiency are crucial to ensuring sustainable development. Thus, identifying the trends and underlying mechanisms of urban expansion is essential to coordinate the rapid pace of urbanization and sustainability in China [4].

Studies have explored determinants of urban expansion, which can be grouped into natural and socioeconomic factors [5,6]. Nature feature, such as geological conditions [7], slope, elevation [8] and climate [9] are considered fundamental for urban development and partly determinate the suitability of construction land [5,6,10]. Socioeconomic factors such as economy growth [11], population growth [12], social process [13,14], infrastructure and transport [5,15,16], national policies and institutions [17,18] are main driving forces. 

However, driving forces of urban expansion in different countries and cities were found to be different. The question is then raised of whether the determinants of urban expansion vary spatially because of the diversity in realistic conditions. China is the world’s largest developing country, and exhibits a variety of physical conditions, levels of economic and social development, and cultures across regions [19]. The land elevation increases gradually from east to west. From the south-eastern coastal regions to north-western inland areas, the distance from the sea becomes greater and precipitation decreases. In southeast China, the vegetation coverage is high and the main landforms are plains, river networks, and hills; by contrast, the main landforms in northwest China are grasslands, deserts, Gobi deserts and snow-covered plateaus. The eastern region also has a relatively high level of economic development because it was at the forefront of the reform and opening-up policy. Chinese population is unevenly distributed and urbanization rates in northeast and eastern regions are higher than those in inland areas [19,20], with 94% of the total population living southeast of the Heihe-Tengchong Line in 2010 [21]. Therefore, China was selected as the research area to verify the variation of determinants of urban expansion.

Some studies have noticed the spatial heterogeneity in influence factors of urban expansion in China. The effects of economic factors and population growth on urban expansion are greater in eastern China than in central and western China [12]. The stimulating effect of road construction is stronger in eastern China than in north-eastern and western China [22]. There are fewer restrictions on urban expansion related to physical conditions in eastern China than in central and western China [5]. However, the influence of regional differences on urban expansion have only been discussed at the regional level or within a single city [5,10]. Identifying the factors influencing urban expansion in different regions can help in understanding the urban process and is essential for ensuring that related regional land use policy decisions are made according to local conditions.

To address this gap in the research, we analysed the spatiotemporal characteristics of urban expansion and identified the effects of spatial heterogeneity in thein the determinants of urban expansion at the prefectural city level in China. This study was conducted as follows. First, the spatiotemporal dynamics of urban expansion in China from 1990 to 2015 were examined. Second, a local regression model, namely the geographically weighted regression (GWR) model, was employed to detect the spatial heterogeneity in the determinants of urban expansion. This study provides insights for forecasting the demand for urban land, optimizing urban systems, and promoting sustainable land use management.

The remainder is structured as follows: Section 2 introduces the materials and methods used, including the study area, datasets, influencing factors, hot spot analysis, and GWR modelling technique. Section 3 introduces the spatiotemporal characteristics of urban expansion from 1990 to 2015 in China. Section 4 presents and discusses the comparison of results of ordinary least squares (OLS) and GWR modelling. Finally, Section 5 presents the conclusions and policy implications based on the results.

## 2. Driving Forces of Urban Expansion and Spatial Heterogeneity in China

### 2.1. General Determinants of China’s Urban Expansion

**Figure 1:**
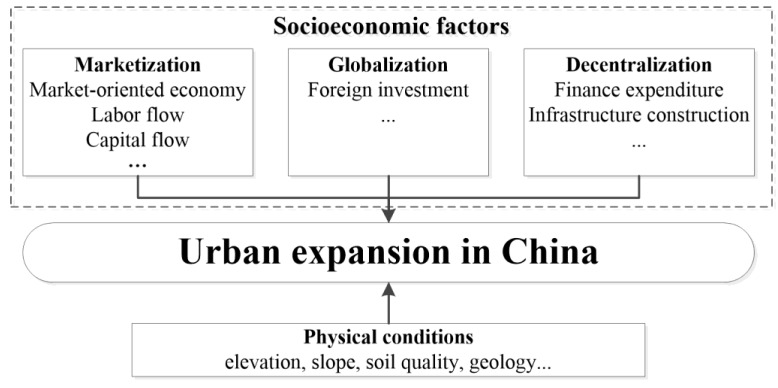
Conceptual framework of the determinants of urban expansion in China.

Many studies have demonstrated that urban expansion, as a product of human activity, is affected by socioeconomic and physical factors [5] (Figure 1). However, socioeconomic factors have a higher frequency, broader and stronger influences than physical factors. Thus, socioeconomic factors are considered the main determinants of urban expansion [15].

Wei has proposed that as China transformed from a socialist to post-socialist society, the triple processes of marketization, globalization, and decentralization promoted regional development in China [23]. Wei’s framework comprehensively explains the mechanisms of urban development and was applied in a serious of case studies that explained regional inequality in China [10,16,24,25]. Therefore, an integrated conceptual framework for this research based on Wei’s analytical framework was developed (Figure 1), and socioeconomic factors can be categorized into marketization, decentralization, and globalization factors.

First, marketization provided a foundation for economic development and urbanization after the implementation of economic reforms [10,26]. The shift from egalitarianism to comparative advantage resulted in unprecedented urban expansion [27]. Marketization and land market reforms transformed funds, labour forces, and land resources into commodities and the market became a major determinant of allocation decisions. Marketization promoted private company development and migration to cities, namely the development of a market-oriented economy and increased labour flows, which increased the demand for land. A market-oriented economy promotes urban expansion directly by increasing the demand for industrial land. In 2014, industrial land, particularly industrial parks, constituted 29.1% of the total state-owned construction land supply [26]. Population migration creates labour resources for industrial development and demands for residential land and infrastructure, which prompts local governments to increase the supply of construction land accordingly. The market-oriented economy and population migration led to the increase of capital flows, particularly investment in fixed assets.

Second, globalization is another major driving force of global urban expansion [10,25], particularly for China [24,28]. Globalization mainly influences urban expansion through foreign investment. China initially attracted foreign investment with its relatively cheap labour and abundant resources. Chinese cities created development zones in the outskirts to attract foreign investment, which promoted urban expansion and spurred ‘development zone fever’ [25]. Moreover, foreign capital was invested in the real estate industry, and this ratio rose from 23.2% in 2011 to 29% in 2014, directly promoting urban expansion [26]. Foreign capital also brought technological innovations into China that improved land use efficiency and inhibited urban sprawl [10].

Third, decentralization indirectly stimulated urban expansion. China's tax reforms of 1994 and land market reforms of 1990 decentralized the central government. The pursuit of economic development and land sales revenue became incentives of urban expansion. The tax reform reduced the proportion of retained tax revenue and forced local governments to develop the economy and collect land-transfer fees to increase revenue. Local governments obtained rural land and leased it for manufacturing purposes or as real estate to increase the local gross domestic product and employment rate. The loss of cultivated land again promoted rural-to-urban migration and increased demand for urban land [10]. Local governments increased financial expenditure on infrastructure construction, such as the establishment of transportation facilities and public service institutions, to attract investment and migrant workers and stimulate the land market [29,30].

In addition, appropriate physical characteristics such as elevation and slope [8] is considered a fundamental condition for urban expansion. This analytical framework systematically integrated socioeconomic and physical factors to explore the various driving forces of marketization, globalization, and decentralization and physical conditions among prefectural cities in China.

### 2.2. Spatial Heterogeneity in the Determinants of Urban Expansion

As the world’s largest developing country, China has spatially uneven natural resource distribution, productive force distribution, and economic and social development levels. The comparative advantages of cities promote urban development and determine urban development patterns. Differences in these urban development patterns have led cities to become specialized, which affects the city scale [31]. Thus, spatial heterogeneity exists in the driving forces of urbanization among cities. China’s globalization process has progressed from eastern coastal to interior regions, and the development in the eastern coastal region led other regions. The market-oriented pattern of urban development was basically formed in eastern coastal regions. Marketization and globalization forces, such as the service industry, foreign capital, and technological and talent development, promoted urban development mainly in eastern coastal regions. The north-eastern region has long been China’s industrial base, and its urban development continues to be industry-driven. Because of the low levels of market development in central and western regions, government intervention through the administrative allocation of resources still greatly influences urban development there. Moreover, located beside the eastern coastal region, the central region may facilitate industry transfers and benefit from the national infrastructure. By contrast, urban development in the western region mainly relies on government support and the primary sector [32]. In addition, many plains are distributed across northern China, whereas southern China is more mountainous. Land resources for urban areas in northern China are abundant and easily lead to inefficient land use and urban sprawl.

## 3. Materials and Methods 

### 3.1. Study Area

**Figure 2:**
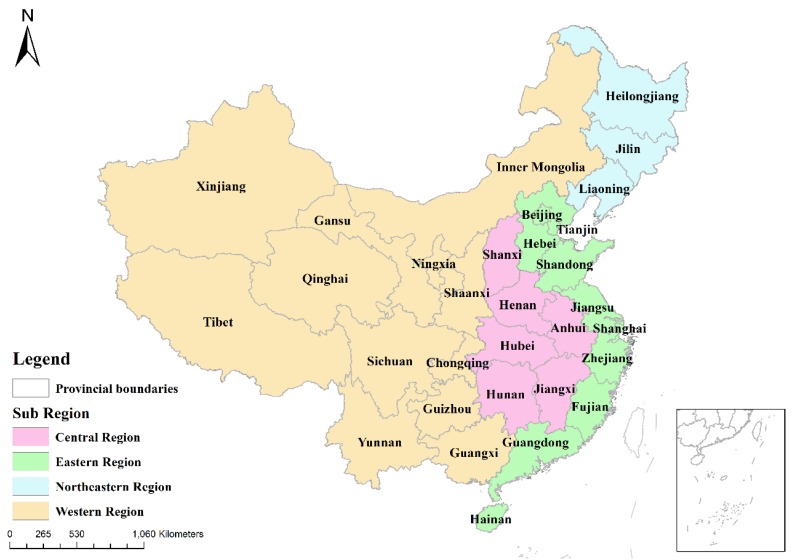
Study area and boundaries of the four sub regions.

Mainland China, the second-largest economy, is the study area. In 1978, the rate of population urbanization was only 17.9%. By 2016, this rate was 57.4%. Based on administrative divisions in 2015, our study area includes 362 prefecture-level cities and 31 provincial-level administrative units. To detect differences in urban expansion, the 31 provinces are grouped into four sub regions based on geographical location and development level, namely the eastern, northeastern, central, and western region [5] (Figure 2).

### 3.2. Data

#### 3.2.1. Land Use Dataset

Images related to six time periods from the China Land Use/Cover Dataset (CLUD), namely the late 1980s (“1990”), 1995, 2000, 2005, 2010 and 2015, were investigated (spatial resolution: 1 × 1 km. The CLUD dataset [33] was analyzed through human–machine interactive interpretation of remote sensing images [17]. The overall accuracy of the CLUD is over 90% for all periods [34]. Built-up land included urban centers, rural settlements, and other types of built-up land, such as mining land, large factories, and large transportation infrastructure located in suburbs. In this study, urban centers and other types of built-up land were classified as urban expansion areas.

#### 3.2.2. Dependent and Independent Variables

The urban expansion scale during 1990–2015 for prefecture and upper-level cities was selected as the dependent variable. According to the conceptual framework (Figure 1), determinants of urban expansion in China can be grouped into socioeconomic and physical factors (Table 1).

*Socioeconomic factors*: Socioeconomic factors includes marketization, globalization and decentralization factors. In terms of marketization, changes of value added of the secondary industry (SND) and value added of the tertiary industry (TRY) were employed to measure the marketization level. Nonagricultural economies boosts urban expansion by increasing urban construction demand [35]. Marketization affects the labour and capital flow [10,27]. Population represents population urbanization, rigid demand, urban market, and agglomeration capability, and is a key indicator for forecasting construction land growth in city master plans. Investment in fixed assets, such as infrastructure construction, renovation, and real estate development, is one of the direct drivers of economic development in China [29,30]. Therefore, we took change of permanent resident population (POP) and total investment in fixed assets (FAI) to represent labour and capital flow.

In terms of globalization, foreign capital actually utilized often finances “land grabs” and promotes urbanization [10,28]. Thus, total amount of foreign capital actually utilized (FC) was selected to measure the influence of economic globalization.

Decentralization affects urban expansion because of local governments’ pursuit for economic growth and land finance [10]. Public financial expenditure relates to a government’s investment in public goods and services, such as affordable housing, transportation systems, and public service facilities [29]. Road construction improves accessibility, thus attracting investment and guiding urban expansion [15,16,36]. Therefore, total public finance expenditure (PFX) and change of area of paved roads in cities (ROD) were selected to represent decentralization. Socioeconomic statistical data were got from China City Statistical Yearbook.

*Physical factors*: As for physical factors, elevation and slope are considered factors that restrict urban expansion, especially in areas with fragile ecological environments and low development levels [8]. Average elevation (ELE) and slope (SLP) were calculated based on SRTM 250 m Digital Elevation Data.

### 3.3. Hot Spot Analysis

The Getis-Ord spatial statistics index ($G_{i}^{*})$ was employed to identify spatial clusters of high values (hot spots) and low values (cold spots) of urban expansion. The $G_{i}^{*}$ statistic is a local spatial autocorrelation index. High values of the $G_{i}^{*}$ statistic denote hot spots where high attribute values were in aggregated distribution, whereas low values denote cold spots where low values were in clumped distribution [29].

The $G_{i}^{*}$ statistic is calculated as:

(1) $$G_{i}^{*}=\frac{\sum_{j=1}^{n}w_{ij}x_{j}}{\sum_{j=1}^{n}x_{j}}$$

where $G_{i}^{*}$ is the $G_{i}^{*}$ statistic of region *i*; $x_{j}$ represents the magnitude of the variable *x* of region *j*; $w_{ij}$ is the spatial weight between region *i* and *j*.

### 3.4. Geographically Weighted Regression (GWR)

Multivariate linear regression techniques include global and local linear regression. Global regression, such as ordinary least squares (OLS), explains the overall correlations by using one equation. Local regression, such as GWR, takes spatial heterogeneity into consideration and estimate different coefficients across regions. GWR can reflect the potential relations among important local variations and is evidently superior to traditional global regression models [19,37]. A GWR model equation is as follows:

(2) $$y_{i}=\beta_{0}\left(u_{i},v_{i}\right)+\overset{n}{\underset{j=1}{\sum }}\beta_{j}\left(u_{i},v_{i}\right)x_{ij}+\varepsilon_{i}$$

where $y_{i}$ is the dependent variable; n is the number of independent variables; $\left(u_{i},v_{i}\right)$ is the location of observation i; $\beta_{j}\left(u_{i},v_{i}\right)$ denotes the j th estimated coefficient at the observation i; $x_{ij}$ is the independent variable; and $\varepsilon_{i}$ is the random error term.

According to Tobler’s first law of geography, objects that are near to each other are more related than those that are not. Therefore, a spatial weight matrix was introduced to denote the relative importance among regions, and the weight value is based on distance [38]. The estimated coefficient can be calculated as follows:

(3) $$\hat{\beta }\left(u_{i},v_{i}\right)={\left(X^{T}W\left(u_{i},v_{i}\right)X\right)}^{-1}\left(X^{T}W\left(u_{i},v_{i}\right)Y\right)$$

where $\hat{\beta }$ is the estimated coefficient of the region i; X is the matrix of independent variables; $X^{T}$ is the transposed form of X; Y is the matrix of dependent variables; and $W\left(u_{i},v_{i}\right)$ is the spatial weight matrix of the *i*-th region.

The choice of the weight value is crucial in the GWR modeling process. The Akaike information criterion (AICc) was used to determine the optimal number of neighboring units. An adaptive bi-square kernel was used to calculate the weight between regions, and the equation is as follows:

(4) $$w_{\mathrm{ij}}=\{\begin{array}{rr} {\left(1-d_{\mathrm{ij}}^{2}/\theta_{i\left(k\right)}\right)}^{2},\; & d_{\mathrm{ij}}<\theta_{i\left(k\right)}\\ 0,\; & d_{\mathrm{ij}}\geq \theta_{i\left(k\right)} \end{array}$$

where $w_{ij}$ the weight value between region i and j in the local model of region i; $d_{ij}$ is the distance between region *i* and *j*; and $\theta_{i\left(k\right)}$ is the adaptive bandwidth.

## 4. Characteristics of China’s Urban Expansion

### 4.1. Dynamic Pattern of Urban Expansion in China, 1990-2015

**Figure 3:**
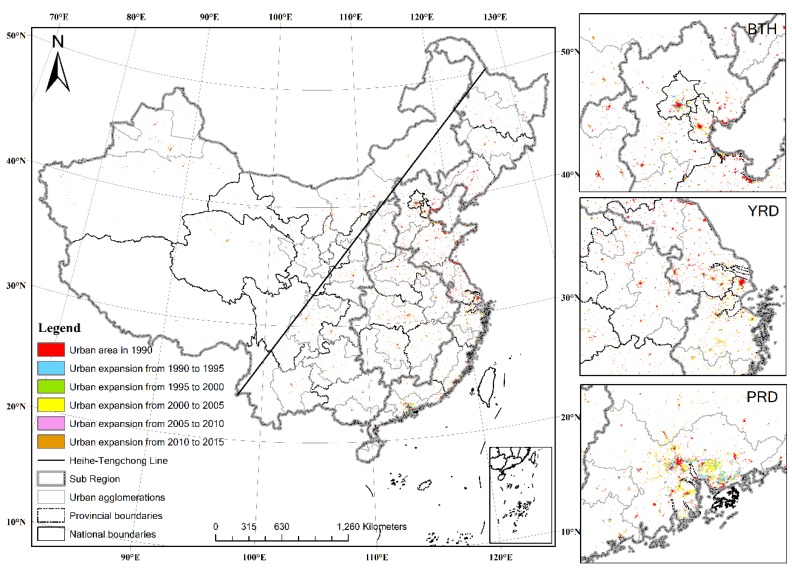
Urban expansion in China during 1990–2015.

Figure 3 shows urban expansion in China during 1990–2015. Urban agglomerations at higher development level, namely Beijing–Tianjin–Hebei (BTH), Yangtze River Delta (YRD) and Pearl River Delta (PRD), where growth rate of urban expansion were high, are displayed individually. The Heihe–Tengchong Line divides China into two approximately equal parts, with 94% of the population living in the eastern part [21]. Urban lands are also mainly distributed in the northeastern of the Heihe–Tengchong Line.

China experienced substantial urban expansion during 1990–2015. The urban land in China grew from 4.88 × 10^4^ km^2^ in 1990 to 1.06 × 10^5^ km^2^ in 2015, a 2.17-fold increase. Urban area accounted for 1.12% of national territory in 2015, and the annual growth rate was 3.15% during 1990–2015 (Table 2).

Annual urban expansion rate and area increased overall in China. The annual increase in urban expansion area during 2010–2015 was the largest, followed by 2000–2005 and 1990–1995. The annual urban expansion rate during 2010–2015 was the highest, followed by 1990–1995 (Table 2).

Urban expansion in China was significantly affected by land use policies and national development strategies. The Decree on Basic Farmland Protection in 1994 and the revised Land Management Law in 1998, which made farmland protection a basic national policy, prevented urban sprawl and loss of cultivated land [17], which is reflected in the smallest and lowest annual urban expansion area and rate, respectively, during 1995–2000. Troughs in annual urban expansion area and rate during 2005–2010 are a consequence of the Global Financial Crisis from 2007 to 2009. The state council announced the Four Trillion Stimulus Package to increase domestic demand and boost economic growth. Therefore, annual urban expansion area and rate saw substantial increases during 2010–2015, especially in central and western regions.

### 4.2. Dynamic Pattern of Urban Expansion in Four Sub Regions of China during 1990-2015

The spatial distribution of urban expansion and urban area in China was aggregated. The global Moran's I of urban expansion across prefecture-level cities during 1990–2015 was 0.32, indicating a significantly positive spatial autocorrelation. Moreover, the global Moran's I of urban area scale across prefecture-level cities in 1990 and 2015 were 0.39 and 0.36, respectively, denoting a significantly positive spatial autocorrelation.

**Figure 4:**
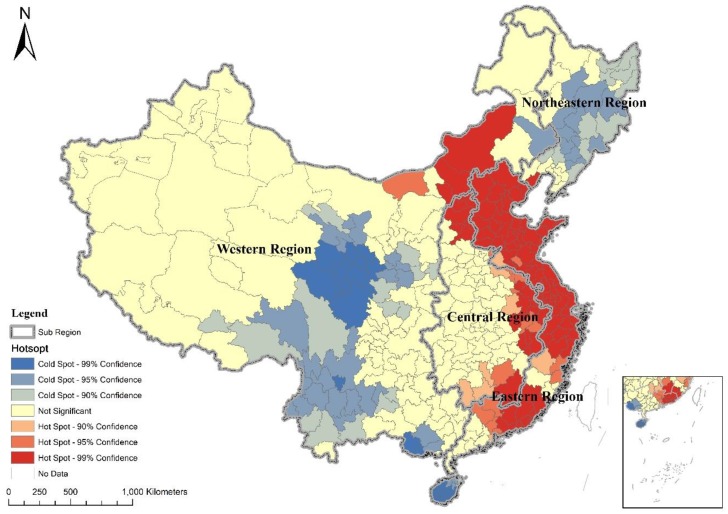
Hot and cold spots of urban expansion in China during 1990–2015.

Urban area and urban expansion during 1990–2015 were mainly distributed in the developed eastern region (Figure 3). Proportions of total urban expansion in the northeastern region and western region were decreasing (Table 2). Hot spots for urban expansion were mainly located in the developed eastern region, whereas cold spots were mainly located in the developing northeastern and western regions (Figure 4). The scale of urban expansion in the eastern region during 1990–2015 was the largest, accounting for 45.85% of total urban expansion. Furthermore, the scale of urban land in the eastern region in 2015 accounted for 46.42% of total urban area. The scale of urban expansion in the northeastern region was the smallest and the growth rate was the lowest. The scale of urban expansion in the northeastern region accounted for 4.76% of total urban expansion, and the proportion of urban area in the northeastern region to total urban area decreased from 12.68% in 1990 to 8.41% in 2015 (Table 2). In response to the Four Trillion Stimulus Package implemented in 2009, urban area increased significantly in the central and western region.

## 5. Modelling Determinants of Urban Expansion in China

Due to adjustments of administrative division and a lack of statistical data, data of 273 prefecture- and upper-level cities were entered into models. The variance inflation factors (VIFs) of the independent variables were all less than 10, and the tolerance values were all greater than 0.1 (Table 3), indicating that independent variables were not collinear.

For urban expansion in China, the adjusted R^2^ (=0.805) was higher and the AICc (=3222.071) was smaller when using GWR than with OLS regression (R^2^ = 0.672, AICc = 3333.053; Table 3). The GWR model outperformed the OLS regression, demonstrating spatial heterogeneity in determinants of urban expansion.

**Figure 5:**
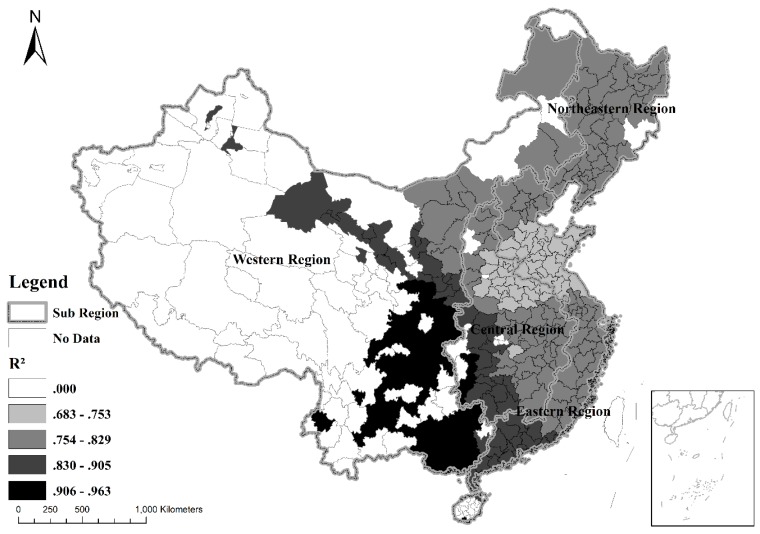
Local R^2^ of the GWR model.

Figure 5 illustrates the spatial distribution of local R^2^ values derived from the GWR model. The blank areas are used to depict cities with no data. The local R^2^ values range from 0.683 to 0.963. The local R^2^ values dropped from the western region to the eastern region (Figure 5). Low R^2^ values denote a poor regression fit in the eastern region, because the open and free market environment in the eastern region [32,37,39] causes urban expansion to be affected by numerous factors.

### 5.1. Socioeconomic Factors

**Figure 6:**
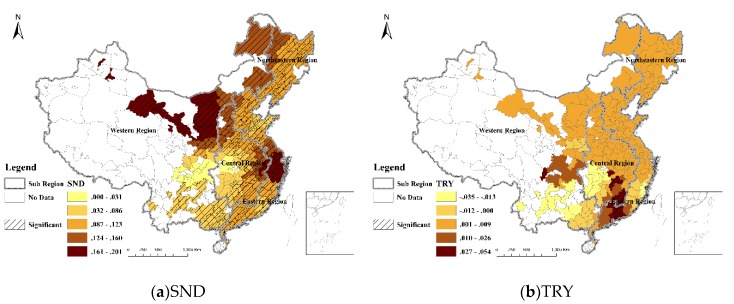

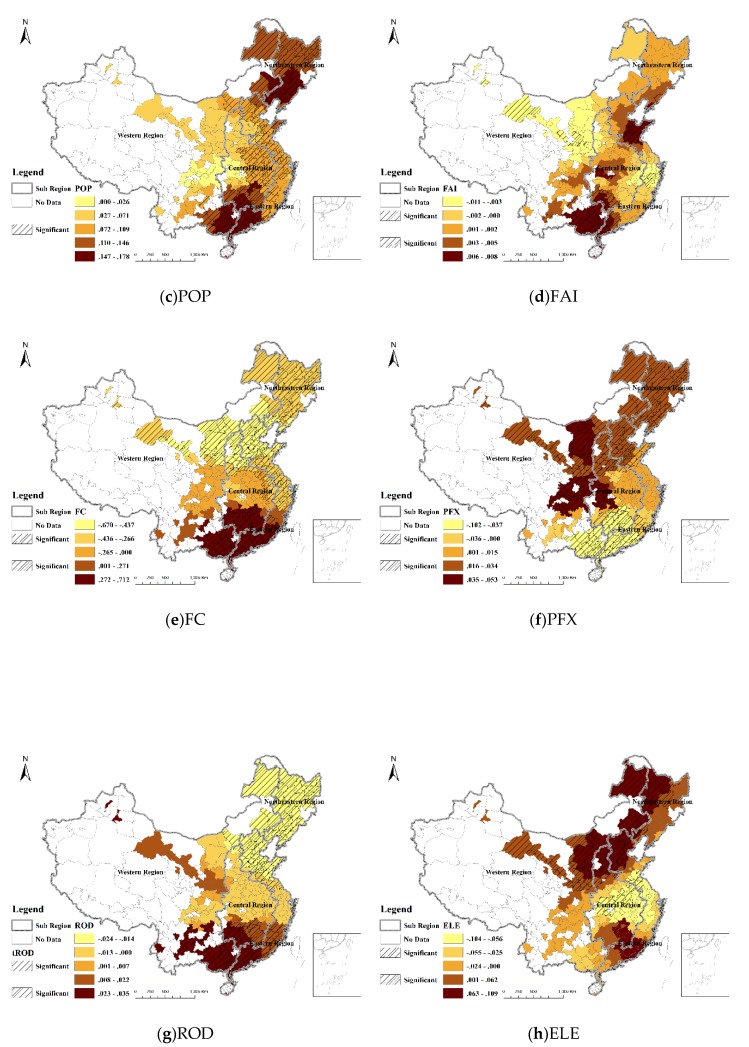

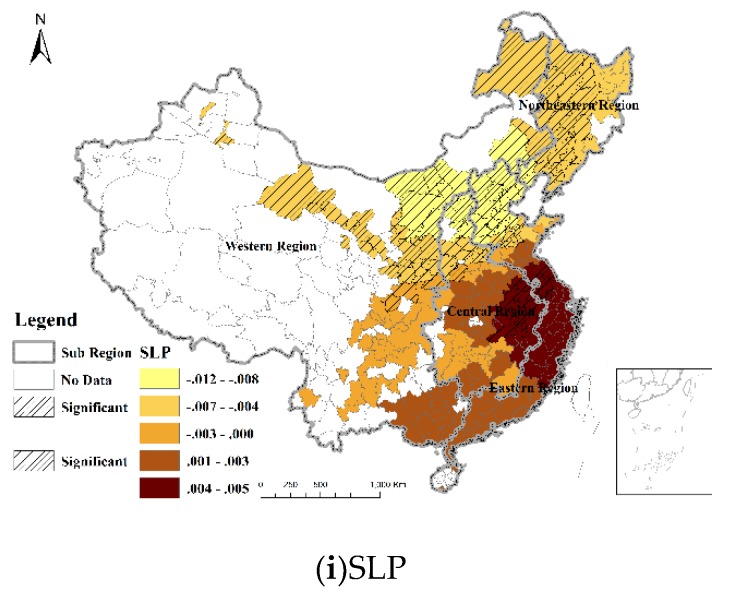
Spatial distribution of the regression coefficients for determinants based on a GWR model.

The growth of added value of the secondary industry (SND) promoted urban expansion significantly. In GWR models, 89.011% of cities demonstrated a significant relation between urban expansion and SND (Figure 6a), and in the OLS regression, the SND was positively and significantly related to urban expansion (Table 3). Coefficients of SND in Yangtze River Delta and northwestern region were the highest and the effects of the SND were larger on cities in the northern region than those in the southern region (Figure 6a).

Tertiary industry (TRY) had limited impact on urban expansion. Only 3.663% of the cities’ urban expansion had a significant relationship with TRY, and they were mainly located in the Pearl River Delta (Figure 6b). In the OLS regression, TRY had a positive but nonsignificant relation with urban expansion (Table 3).

The growth of the permanent resident population (POP) can explain 63.736% of the cities’ urban expansion, which were mainly in the eastern region, northeastern region, and southeastern part of central region. Urban expansion in the western region and the western part of central region was not significantly correlated with permanent resident population growth. (Figure 6c). In the OLS regression, POP was significantly and positively related to urban expansion (Table 3).

Investment in fixed assets (FAI) had significant bidirectional-impact on urban expansion in 22.711% of cities. FAI had a significantly positive relation with urban expansion in cities located on the Shandong Peninsula and in the southwestern region (80.645%). By contrast, in cities located in the northwestern region and Zhejiang Province, FAI had a significantly negative relation (19.355%) (Fig 6d). In the OLS regression, FAI was significantly and positively related to urban expansion (Table 3).

The variable of actually utilized foreign capital (FC) was found to have a significant bidirectional-effect on 73.993% of studied cities’ urban expansion, displaying a significantly negative relation in the northern and Yangtze River Delta regions (72.277%). By contrast, cities in the Pan-Pearl River Delta region had a significantly positive relation (27.723%) with FC (Figure 6e). In the OLS regression, FC was significantly and negatively related to urban expansion (Table 3).

The variable of total public finance expenditure (PFX) can explain 72.161% of studied cities’ urban expansion, showing a significant positive relation in the northern region (72.081%). By contrast, it had a significantly negative relation in the Pan-Pearl River Delta region (27.919%; Figure 6f). In the OLS regression, PFX was significantly and positively related to urban expansion (Table 3).

Road construction (ROD) can explain 51.648% of urban expansion in the cities studied, displaying a significant positive relation in the Pan-Pearl River Delta region (49.645%). By contrast, it had a significantly negative relation with urban expansion in the northeastern and Bohai Rim regions (50.355%; Figure 6g). In the OLS regression, ROD was significantly and positively related to urban expansion (Table 3).

### 5.2. Physical Factors

Only 32.601% of cities were found a significant relation between elevation (ELE) and urban expansion. ELE had a significantly positive relation with such expansion in the northwestern and northeastern region (79.775%) and a significantly negative relation in cities located on the Yangtze Plain and in the southern part of the North China Plain (32.601%; Figure 6h). In the OLS regression, ELE had a positive but nonsignificant correlation with urban expansion (Table 3).

Slope (SLOPE) can explain 42.857% of the urban expansion in the cities studied, having a significantly negative relation with urban expansion in the northern region (82.906%) (Figure 6i). While in the OLS regression, SLOPE had a negative but nonsignificant relation with urban expansion (Table 3).

## 6. Discussion

### 6.1. Socioeconomic Factors

#### 6.1.1. Marketization

Previous studies generally used GDP to represent the industry development [5,24], while we divided into secondary industry and tertiary industry. Previous studies considered secondary industry growth as a positive factor for urban expansion [15,40], and GWR results showed that urban expansion had significantly positive relationship with SND in most cities. The effects of the SND were larger on cities in the northern region than those in the southern region (Figure 6a). As the traditional industrial center of China, the northern region had a superior industrial base and SND development was one of the major driving forces of urbanization. By contrast, the industrial base in southern region was relatively weak, and the SND played a relatively small role in promoting urban growth.

The growth of the tertiary industry can shift the economic development pattern from land-intensive industries to service industries with relatively little dependence on land resources. Moreover, the development of tertiary industry improves residents’ lifestyles, thereby promoting their demand for green open spaces and suburban ecological land. This leads to central and local governments devoting more resources to protecting and restoring ecological land [15]. According to the results of GWR model and OLS model, urban expansion was least affected by TRY. Previous studies even identified a negative relation between tertiary industry development and urban expansion generally [15,41], while a positive correlation was found in the Pearl River Delta region. Because the development level of tertiary industry in the Pearl River Delta region is high and urban development is significantly driven by tertiary industry [36].

Population growth significantly promoted urban expansion generally [5,12,29,30,41], while in GWR model, urban expansion in the western region and the western part of central region was not significantly affected by permanent resident population growth (POP). Due to the relatively low level of economic development there, people migrate to work in the eastern developed areas, which leads to a low growth rate of the permanent resident population [42]. The low level of urbanization in this region also led to a low growth rate of urban residents.

Investment in fixed assets (FAI) was identified as one of main driver of urban expansion [15,29,30]. While a negative relation between FAI and urban expansion was found in the northwestern region and Zhejiang Province, because land use management is extensive and investment intensity is relatively low in the northwestern region. The existing urban land can absorb new investment in fixed assets [41]. However, because Zhejiang Province has entered the later industrialization or developed stage [29,30,43], the imbalance between the supply and demand of land resources gradually emerged. Intensive land use and growth are valued. Investment in fixed assets is used to explore urban land use potential, such as urban renewal projects, which helps to reduce the consumption of land resources and promote land use efficiency [41]. Therefore, reasons for the negative correlation between urban expansion and FAI in the northwestern region and Zhejiang Province are different.

Marketization factors have a substantial effect on urban expansion generally. However, spatial heterogeneity in the strength and direction of marketization factors (SND, TRY, POP, FAI) at the local scale was obvious. The secondary industry(SND)and permanent resident population (POP) are crucial factors for promoting urban expansion, especially in the developed eastern and southern regions. The tertiary industry (TRY) had no effect on urban expansion in general, and only promoted urban expansion in the developed Pearl River Delta region. Secondary industry is highly dependent on resource input, while tertiary industry is environmental friendly and can promoting land use efficiency. Thus, for the demand of economic growth transition, local governments and policymakers should formulate corresponding policies to attract tertiary industry. Investment in fixed assets (FAI) was found to have different impacts in different regions.

#### 6.1.2. Globalization

In the GWR model, foreign capital (FC) was found to act as both incentive and disincentive to urban expansion. Foreign capital was identified to promote urban expansion in Guangzhou [28], while foreign capital showed a negative correlation with urban expansion in the national model [39]. FC had bidirectional-impact on urban expansion. This is because compared with other urbanization modes, the land use efficiency of foreign-capital-driven urbanization is high. Foreign enterprises are profit-oriented and thus cost saving related to land is an important factor to be considered in their investments. By contrast, state-owned enterprises’ lands are allocated by local government, and many small-scale private enterprises and township enterprises acquire lands without the approval of the administrative authority. The phenomena of inefficient use and wastage of land is a serious concern. Studies have found that after a series of policy variables are added, the effect of foreign-capital-driven urbanization on land conservation is no longer significant. This is because most foreign investment is distributed in cities with policy advantages and high levels of marketization. Therefore, the impact of foreign investment on urban expansion can be seen as arising from the combined influences of urban policy, management, and foreign investment [40]. As Yangtze River Delta region is located in the Middle-Lower Yangtze plain and the northern region has the Northeast China plain, land resources there are abundant and economic development there depended heavily on state-owned enterprises. Land use efficiency is low. Foreign investment can improve land-use efficiency and inhibit urban sprawl. However, land resources in the Pan-Pearl River Delta region is poor because it is located in Southern Hills. The Pan-Pearl River Delta region is at the forefront of economic reform [37], and economic development there depended heavily on foreign enterprises. Thus, foreign capital promotes urban expansion.

#### 6.1.3. Decentralization

Public finance expenditure (PFX) had a significant bidirectional-effect on urban expansion. In previous studies, PFX was identified as a positive factor for urban expansion generally [29,30], while in GWR model, PFX exhibited a positive correlation only in the northern region and a negative correlation in the Pan-Pearl River Delta region, where economic development has entered a late stage of industrialization [29,30,43], is predominantly mountainous and hilly. The imbalance between the supply and demand of land resources is a pertinent concern. Public finances were invested in the redevelopment of stock land, such urban renewal projects, to improve land-use efficiency instead of increasing available land.

Road construction (ROD) was identified to have a significant bidirectional-effect on urban expansion, while previous studies identified ROD as a positive factor for urban expansion [5,15,16]. A positive relation between ROD and urban expansion was identified in the developed Pan-Pearl River Delta region and a negative correlation northeastern and Bohai Rim regions. Because after China entered a period of economic transition, the traditional industries in the northeastern region went into decline, leading to urban shrinkage and population loss. Road construction improves accessibility and makes travel more convenient [5,15,16], accelerating population decline in this region.

Decentralization factors have different effects in the southern and northern regions. Public financial expenditure had a bidirectional effect, and the direction of the effect depended on the relation between the supply and demand of land resources. PFX was found to inhibit urban expansion in the Pan-Pearl River Delta region, with a shortage of land resources, and promote urban expansion in the northern region, with abundant land resources. ROD also had a bidirectional effect on urban expansion based on the developmental stages of regions. Road construction may have accelerated population loss and slow down urban expansion to some extent in the northeastern region, where economic development experienced a bottleneck. By contrast, Road construction promoted urban expansion in the developed Pan-Pearl River Delta region and effectively guided urban expansion. Therefore, rational designing road networks and infrastructure system is of great value to guide a healthy urban development.

### 6.2. Physical Factors

Elevation was found to have limited impact on urban expansion in GWR model. Previous generally identified a negative correlation between ELE and urban expansion [5], while a positive correlation was found in the northwestern and northeastern region. Because the ecology of the northwestern region is fragile, and the northeastern region is an important grain-producing area in China. The success in protection of high-quality farmland [17,44] can partly explain the negative relation, because high-quality farmlands are mainly distributed at low altitude areas [5]. Moreover, regionally balanced development strategies such as Western Region Development and Revitalization of the Northeastern Region, increased the supply of construction land in these high-altitude areas and promoted rapid urban expansion [15].

Slope (SLOPE) showed a negative correlation with urban expansion generally, and previous generally identified slope as a negative factor for urban expansion generally [5,15], while the constraining effect of slopes was not significant in most parts of the southern region.

Overall, the constraining effect of physical factors, including elevation (ELE) and slope (SLOPE), was limited. The constraining effect of physical conditions was found weakened as urbanization level increased [5]. In GWR model, the effect of physical factors on the developing northern region was greater than that in the developed southern region.

### 6.3. Limitations

Studies on identifying explanatory factors of urban expansion are fruitful [6,16,22]. A series of factors were selected based on the comprehensive Wei’s analytical framework [23]. However, due to the research scale and a lack of available data, it was not possible to study some factors, and urban landscape planning is one of them. Urban landscape planning is considered as an effective tool to shape city development and urban expansion, but most such studies focused on a single city or several cities at a relatively micro scale [45,46]. Because landscape planning arranges land use layouts specifically, and it is appropriate to evaluate its effectiveness in a single city or a small region. While this paper studied 273 cities at a macro scale and focused on the impact of macro-economic and social factors on urban expansion. Furthermore, we will continue this study and select several typical cities to investigate micro factors such urban landscape planning, building restrictions and social problems. At last, nighttime lights have been used to detect global urban expansion and we can use GWR model to identify the varying driving forces of global urban expansion in the future.

## 7. Conclusions and Policy Implications

To fill the research gap in identifying the varying determinants of urban expansion among prefecture-level cities nationwide, we first explored the spatiotemporal characteristics of China’s urban expansion from 1990 to 2015 to identify the variations in determinants of urban expansion among prefecture-level cities. We then compared the results of the OLS and GWR models. The GWR model outperformed the OLS model, which confirmed the spatial heterogeneity of the determinants of urban expansion.

Urban areas expanded dramatically in China from 1990 to 2015, increasing 2.17-fold from 4.88 × 10^4^ km^2^ in 1990 to 1.06 × 10^5^ km^2^ in 2015. Urban expansion in China was uneven and substantially influenced by land use policies and national development strategies. Urban areas were mainly distributed in the eastern coastal area, whereas the scale of urban expansion in the north-eastern and south-western regions was small.

Socioeconomic and physical factors considerably affected urban expansion in China. The impact of socioeconomic transition can be conceptualized into transition of marketization, globalization, and decentralization. Marketization stimulated urban expansion by accelerating labour and capital flows. Globalization triggered urban expansion by attracting foreign investment. Decentralization prompted local governments to develop local economies and pursue revenue through land leasing.

A fine-scale GWR model identified evident spatial heterogeneity in strength and direction of several determinants (SND, TRY, POP, FAI, FC, PFX, ROD, ELE, SLP) at the prefectural city level nationwide. Marketization played a vital role in urban expansion and had a stronger impact in the developed eastern and southern regions than in the developing northern and western regions. Secondary industry (SND) and permanent resident population (POP) were two major drivers of urban expansion in China, and their correlation with urban expansion was stronger in the developed eastern and southern regions. However, the tertiary industry (TRY) only positively affected urban expansion in the developed Pearl River Delta. Fixed asset investment (FAI) was positively related to urban expansion within parts of Chinese cities. The influence of globalization bi-directionally affected urban expansion. Because foreign firms are profit-oriented, foreign capital (FC) inhibited urban sprawl in the less-developed western and northern regions and increased urban areas in the developed Pan-Pearl River Delta region. Decentralization also bi-directionally affected urban expansion. The direction of the effect of public financial expenditure (PFX) depended on whether the relationship between the supply and demand of land was balanced, whereas that of road construction (ROD) depended on the developmental stage of the region. The constraining effect of physical factors such as elevation (ELE) and slope (SLOPE), was limited and exerted a greater effect on the developing northern region than on the developed southern region.

China’s decision-makers and urban planners should implement locally oriented policies for urban planning and urban land management because regions differ regarding the determinants that promote or inhibit urban expansion. (1) SND and POP did not substantially correlate with urban expansion in parts of the developing western region, and the TRY did not substantially affect urban expansion in most areas. Consequently, strict land use standards, including those based on investment, output value, and population per unit of construction land in urban planning, should be established to maximize land use efficiency, particularly in the western region where economic growth relies on extensive urban land use. The growth of urban populations and non-agricultural industries were not synchronized with urban expansion. Strengthening land use standards can help to transform economic growth from an extensive to intensive pattern. Corresponding policies to attract tertiary industry could be made to accelerate economic transition and promote land use efficiency. (2) FAI and PFX promoted urban expansion in developing areas and inhibited urban expansion in developed regions because the proportion of capital devoted to the redevelopment of stock land to improve land use efficiency was higher. Therefore, governments of developing areas should optimize the structure of investment in fixed assets and public financial expenditure. The proportion of industries reliant on land, such as the real estate industry, should be reduced to curb investment-driven urban sprawl. Moreover, an urban renewal planning or a topic about urban renewal in urban master planning are needed to guide the redevelopment of stock land. (3) The regression coefficients of ROD were positive in developed southern regions and negative in developing northern regions. Infrastructure construction increased the demand for construction land in southern regions while accelerating population migration to developed southern regions in northern regions. Thus, the government should prevent excessive land occupation for infrastructure construction in southern regions and inefficient duplication of infrastructure systems in northern regions when developing urban planning. Furthermore, rational designing road networks is key to guiding the healthy development of cities. (4) The constraining effects of natural conditions have weakened, particularly in the developed southern region, and the ecological environment in the western region is fragile. Urban growth has directly affected ecological safety. Therefore, the government should strengthen the protection of key ecological zones to promote environmentally friendly development. As for landscape planning, one of the priority is to protect ecological environment. Therefore, fragile ecological regions should be protected and systematic ecological space should be planned in landscape planning.

## Figures and Tables

**Table 1:** Summary of variables.

Category		Abbreviation	Definition
Dependent variable		UE	Urban expansion scale (1990–2015) (km^2^)
Socioeconomic factors	Marketization	SND	Change of value added of secondary industry (1990–2015) (RMB 1000 million)
		TRY	Change of value added of tertiary industry (1990–2015) (RMB 1000 million)
		POP	Change of permanent resident population (1990–2015) (million)
		FAI	Total investment in fixed assets (1990–2015) (RMB 1000 million)
	Globalization	FC	Total amount of foreign capital actually utilized (1990–2015) (US$1000 million)
	Decentralization	PFX	Total public finance expenditure (1990–2015) (RMB 1000 million)
		ROD	Change of area of paved roads in cities (1990–2015) (km^2^)
Physical factors		ELE	Average elevation (m)
		SLP	Average slope (%)

**Table 2:** Urban area and urban expansion in China during 1990–2015.

Indicators	Period	National	Eastern Region	Northeastern Region	Central Region	Western Region
Urban area (km^2^)	1990	48796	22974	6188	9617	10017
	1995	60314	29723	6928	11731	11932
	2000	65694	32203	7292	13102	13097
	2005	77330	40140	7558	14866	14766
	2010	84920	44412	7934	16454	16120
	2015	105840	49131	8905	21610	26194
Proportion of total urban expansion (%)	1990	100	47.08	12.68	19.71	20.53
	1995	100	49.28	11.49	19.45	19.78
	2000	100	49.02	11.10	19.94	19.94
	2005	100	51.91	9.77	19.22	19.09
	2010	100	52.30	9.34	19.38	18.98
	2015	100	46.42	8.41	20.42	24.75
Annual urban expansion area (km^2^/yr)	1990–1995	2303.6	1349.8	148	422.8	383
	1995–2000	1076	496	72.8	274.2	233
	2000–2005	2327.2	1587.4	53.2	352.8	333.8
	2005–2010	1518	854.4	75.2	317.6	270.8
	2010–2015	4184	943.8	194.2	1031.2	2014.8
	1990–2015	2281.76	1046.28	108.68	479.72	647.08
Annual urban expansion rate (%)	1990–1995	4.33	5.29	2.28	4.05	3.56
	1995–2000	1.72	1.62	1.03	2.24	1.88
	2000–2005	3.32	4.50	0.72	2.56	2.43
	2005–2010	1.89	2.04	0.98	2.05	1.77
	2010–2015	4.50	2.04	2.34	5.60	10.20
	1990–2015	3.15	3.09	1.47	3.29	3.92

**Table 3:** Results of the OLS regression and GWR.

	OLS				GWR		
	Coefficient	Standardized coefficient	VIF	Tolerance	Sig. (*P* < 0.10)	+	－
SND	0.092 ***	0.638	8.316	0.120	89.011%	100.000%	0.000%
TRY	0.002	0.036	1.664	0.601	3.663%	100.000%	0.000%
POP	0.102 ***	0.128	1.620	0.617	63.736%	100.000%	0.000%
FAI	0.002 *	0.118	8.161	0.123	22.711%	80.645%	19.355%
FC	−0.236 ***	−0.276	7.180	0.139	73.993%	27.723%	72.277%
PFX	0.006*	0.128	5.820	0.172	72.161%	72.081%	27.919%
ROD	0.011**	0.136	3.132	0.319	51.648%	49.645%	50.355%
ELE	0.007	0.021	1.451	0.689	32.601%	79.775%	20.225%
SLP	−0.001	−0.040	1.425	0.702	42.857%	17.094%	82.906%
Constant	33.030	/	/	/	/	/	/
AICc	3333.053				3222.071		
Adjusted R square	0.672				0.805		

Note: * *p* < 0.1, ** *p* < 0.05, *** *p* < 0.01.
